# IGHV gene mutational status and 17p deletion are independent molecular predictors in a comprehensive clinical-biological prognostic model for overall survival prediction in chronic lymphocytic leukemia

**DOI:** 10.1186/1479-5876-10-18

**Published:** 2012-01-30

**Authors:** Pietro Bulian, Davide Rossi, Francesco Forconi, Giovanni Del Poeta, Francesco Bertoni, Emanuele Zucca, Marco Montillo, Gabriele Pozzato, Giovanni D'Arena, Dimitar G Efremov, Roberto Marasca, Francesco Lauria, Gianluca Gaidano, Valter Gattei, Luca Laurenti

**Affiliations:** 1 Clinical and Experimental Onco-Hematology Unit, IRCCS Centro di Riferimento Oncologico, via Franco Gallini 2, 33170 Aviano (PN), Italy; 2 Division of Hematology, Amedeo Avogadro University of Eastern Piedmont, Novara, Italy; 3 Division of Hematology and Transplant, University of Siena, Siena, Italy; 4 Hematology, Tor Vergata University, Rome, Italy; 5 Laboratory of Experimental Oncology and Lymphoma Unit, Oncology Institute of Southern Switzerland (IOSI), Bellinzona, Switzerland; 6 Department of Oncology/Hematology, Niguarda Ca'Granda Hospital, Milan, Italy; 7 Department of Internal Medicine and Haematology, Maggiore Hospital, Trieste, Italy; 8 Onco-Hematology Department, IRCCS Centro di Riferimento Oncologico della Basilicata, Rionero in Vulture (Pz), Italy; 9 CNR Campus "A. Buzzati-Traverso", ICGEB Outstation-Monterotondo, Rome, Italy; 10 Department of Medical Sciences, Section of Internal Medicine and Hematology, University of Modena and Reggio Emilia, Modena, Italy; 11 Institute of Haematology, Catholic University of the Sacred Heart, Rome, Italy

**Keywords:** Chronic lymphocytic leukaemia, Prognosis, Prognostic score, Nomogram

## Abstract

**Background:**

Prognostic index for survival estimation by clinical-demographic variables were previously proposed in chronic lymphocytic leukemia (CLL) patients. Our objective was to test in a large retrospective cohort of CLL patients the prognostic power of biological and clinical-demographic variable in a comprehensive multivariate model. A new prognostic index was proposed.

**Methods:**

Overall survival and time to treatment in 620 untreated CLL patients were analyzed retrospectively to evaluate the multivariate independence and predictive power of mutational status of immunoglobulin heavy chain variable gene segments (IGHV), high-risk chromosomal aberration such as 17p or 11q deletions, CD38 and ZAP-70 expression, age, gender, Binet stage, β2-microglobulin levels, absolute lymphocyte count and number of lymph node regions.

**Results:**

IGHV mutational status and 17p deletion were the sole biological variables with independent prognostic relevance in a multivariate model for overall survival, which included easily measurable clinical parameters (Binet staging, β2-microglobulin levels) and demographics (age and gender). Analysis of time to treatment in Binet A patients below 70 years of age showed that IGHV was the most important predictor. A novel 6-variable clinical-biological prognostic index was developed and internally validated, which assigned 3 points for Binet C stage, 2 points/each for Binet B stage and for age > 65 years, 1 point/each for male gender, high β2-microglobulin levels, presence of an unmutated IGHV gene status or 17p deletion. Patients were classified at low-risk (score = 0-1; 21%), intermediate-risk (score 2-5; 63% of cases), high-risk (score 6-9; 16% of cases). Projected 5-year overall survival was 98%, 90% and 58% in low-, intermediate- and high-risk groups, respectively. A nomogram for individual patient survival estimation was also proposed.

**Conclusions:**

Data indicate that IGHV mutational status and 17p deletion may be integrated with clinical-demographic variables in new prognostic tools to estimate overall survival.

## Background

According to the updated National Cancer Institute-Working Group (NCI-WG) guidelines, indication for treatment of chronic lymphocytic leukemia (CLL) still depends on clinical stage and disease activity [1]. In this context, measurements of biological prognostic markers, namely CD38, ZAP-70, mutational status of immunoglobulin heavy chain variable gene segments (IGHV), are judged as mandatory in the context of clinical trials, but not in general practice, since they fail to influence therapeutic decisions [1]. The only exception is represented by analyses of chromosomal aberrations by interphase fluorescence in-situ hybridization (FISH), given the presence of high-risk cytogenetic lesions (del11q and del17p), which may predict resistance to chemotherapy-based treatments [2]. Wierda et al. [3] proposed to combine a set of clinical risk factors, i.e age, gender, Rai staging, absolute lymphocyte count (ALC) and number of involved lymph node regions (LNR), with an inexpensive and widely available serum marker such as beta2-microglobulin (β2 M) to develop a prognostic index (PI) stratifying patients in three risk groups with different expected median survival, and a nomogram, estimating individual patient survivals. This model was subsequently validated in independent patients series also using time to first treatment as end-point [4-8]. A reduction of this model from six to four variables, i.e. age, gender, β_2 _M levels and Binet staging, was also shown to predict survival with equal or even better performance [8]. The object of the present study was to provide evidence that prognostic models for overall survival based on clinical variables [4-8] could be improved by information on biological risk factors. By retrospectively analyzing a multicentre CLL population of over 600 untreated patients the most significant and independent biological and clinical prognosticators were integrated in a new clinical-biological prognostic index for group stratification and in a novel nomogram for estimating individual survival.

## Methods

### Patient population

Between 1996 and 2008 a cohort of 620 CLL patients was collected in the context of a larger multicenter patient dataset (n = 1037), previously utilized to propose a modified prognostic model and nomogram [8], according to the availability of the following biological prognosticators: IGHV mutational status, chromosomal abnormalities, as detected by interphase FISH, and flow cytometric expression of CD38 and ZAP-70. Moreover, since most of the diagnoses of the original patient set were made before the publication of the revised NCI-WG guidelines [1], all cases of previously defined CLL that could be re-classified as monoclonal B cell lymphocytosis (MBL) were removed accordingly. The percentage of recruited cases in the different centers was: 30% at Roma Catholic University, 25% at Novara, 15% at Roma Tor Vergata, 8% at Siena, 6% at Milano, 4% each in the other 4 centers. Cut-points for LNR were as previously reported [3]. Continuous variables age and β_2 _M levels were categorized using cut-points at 65 years for age and at the upper limit of normal (ULN) for β_2 _M, as deduced by the analysis of martingale residuals plots [9]; ALC was categorized at the median, since the martingale residual plots did not show any suitable cut-point.

### Biological prognosticators

Evaluation of biological prognosticators was centralized in few reference laboratories, utilizing previously validated common procedures; in detail, 5 centers performed IGVH mutational analysis, 6 centers performed cytogenetics and flow cytometry. IGHV mutational status was performed as previously reported [10]. Cytogenetic abnormalities involving chromosomes 11 (del11q22; hereafter del11), 12 (trisomy 12), 13 (13q14.3) and 17 (del17p13; hereafter del17) were investigated by interphase FISH, as reported [11]. Results of FISH analyses were classified as unfavourable when high-risk genomic aberrations (del17p and or del 11q) were present [12-14]. ZAP-70 measurements were determined by flow cytometry, utilizing the 20% of positive CLL cells as cut-off to discriminate between ZAP-70 positive and negative cases [15-18]. CD38 measurements were performed as reported [19], using a threshold at 30% expression to define positive cases. All the variables were measured at or within one year from diagnosis and always before treatment on either fresh or frozen samples. Data were used upon informed consent from patients and approval by Institutional Review Boards (Centro di Riferimento Oncologico, Aviano; Catholic University of the Sacred Heart, Rome), and in accordance with the Declaration of Helsinki.

### Statistical methods

All analyses were performed in R, an open source statistical package (http://www.r-project.org/). Median follow-up was computed using the reverse censoring method. The primary end points were overall survival (OS) and time-to-first-treatment (TTT), defined as described [1,20,21]. OS was estimated using Kaplan-Meier plots and compared between groups by log-rank test. Univariate and multivariate Cox models were used to verify independent prognostic power of each parameter. Model minimization was performed by stepwise backward elimination. A *p *value < 0.05 was considered to be statistically significant. Departure from proportionality in hazard was tested in all Cox models. The predictive accuracy of various Cox models was evaluated by calculating the concordance index (c-index), which is a probability of concordance between predicted and observed survival, equal to the area under the receiver operating characteristics curve for censored data [22]. A c-index of 0.5 indicates that outcomes are completely random, whereas a c-index of 1 indicates that the model is a perfect predictor. Prediction error was calculated as 1-c-index. U-statistics was applied to test the significance between different c-index values [22]. Nomogram was developed and calibrated following published methods [22]. Final risk group scoring was developed in four step: 1. selection of independent predictive variables; 2. fitting of a Cox model with selected variables; 3. score assignments based on regression coefficients; 4. identification of best cut-point to split the score in 3 risk groups by recursive partitioning [23]. Internal validation for step 1. and 2. was performed with bootstrap .632+ method [24,25] with B = 620 bootstrap samples and (step 2) with cross-validation [26]. Variables selected with a frequency greater than 50% were entered in the final model. Risk score categorical model obtained by recursive partitioning was internally validated by bootstrap methods applied to tree-based analysis [27]. Finally, the whole model building procedure was validated by a comprehensive leave-one-out cross validation (see Additional file 1: supplementary statistical methods). All p values are based on two-tailed tests.

## Results

### Patients characteristics

**Figure 1:**
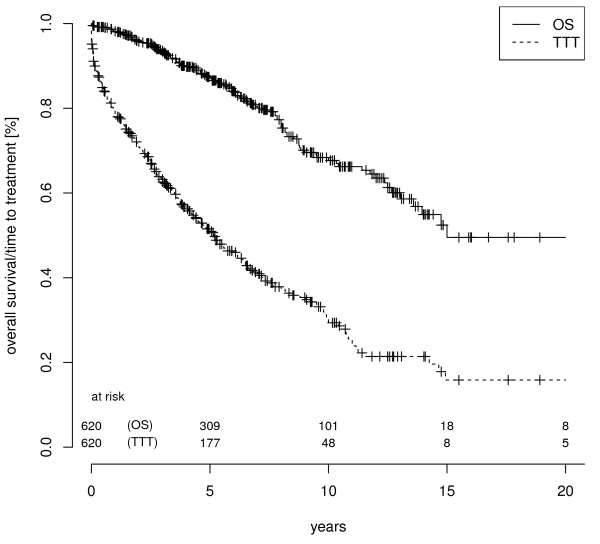
**Overall survival (OS) and time to treatment (TTT) in the whole cohort of 620 CLL patients**.

Patients characteristics are reported in Table 1. Treatment was administered according to NCI-WG indications. Deaths occurred mostly in treated patients (83%). Deaths among untreated patients aged beyond 70 years accounted for 11% of all deaths. All patients characteristics were balanced across age groups <55, 55-64, 65-4 and ≥ 75(chi-square tests), except for a greater proportion of males in the <55 age group and a greater proportion of high β2 M levels and deaths events in the ≥75 age group. Kaplan-Meyer plots of OS and TTT are shown in Figure 1.

**Table 1:** Patients characteristics (n = 620)

median age, years (range)	65 (21-92)
median ALC, x10^9^/L (range)	14 (2-460)

median β_2_M, xULN (range)	1.06 (0.13-11.9)

LNR ≥3	25%

Rai stage	

0	48%

I-II	44%

III-IV	8%

Binet stage	

A	70%

B	22%

C	8%

Male sex	60%

CD38 expression >30%	27%

ZAP-70 expression >20%	41%

Unmutated IgVH	41%

del11q-	9%

del17p-	10%

Year of diagnosis	

<=2000	29%

2001-2005	49%

>2005	22%

Treated	53%

median TTT, years	5.2

Chemotherapy	28%

Chemoimmunotherapy	15%

missing data	10%

Dead	20%

median OS, years	15

ALC, absolute lymphocyte count; LNR, number of lymph node involved regions; ULN, upper limit of normal; β_2 _M, beta-2 microglobulin, OS, overall survival; TTT, time to treatment. Complete data (n = 620) available for all variables, except for therapy

### Univariate and multivariate analysis for OS and TTT

In univariate analysis for OS all clinical and biological variables were significant, except for del11q (Table 2). The effect of chemoimmunotherapy was small, without statistical significance. The effect of period of diagnosis was significant, with patients diagnosed 2000-2005 and >2005 at increasing risk compared to patients diagnosed before 2001. Based on these results, multivariate analyses were adjusted for the year of diagnosis introduced in the model as a three level stratification factor (<=2000, 2001-2005, >2005). Age dependent variations of variables effects were explored by including an age interaction term either in continuous form or as a four-group (<55, 55-64, 65-74, ≥75) ordinal variable in multivariate models. No significant variations in hazard ratio (HR) values were found (*p *> 0.05 for all interactions). The selection of the variables entered in final model was internally validated by bootstrap .632+ method [24]. All the variables introduced in the final model were selected in more than 50% of bootstrap samples (Table 3); prediction error in this step of model building was 0.244. The final model fitting was also validated by bootstrap .632+ method, showing at this step a prediction error of 0.247. Leave-one-out cross validation [24,26]. showed that neither β_2 _M nor gender, the least important variables, could be safely removed from the model (Table 4). Univariate and multivariate analyses of TTT, performed on the subset of Binet A patients below 70 years of age, are shown in Table 5.

**Table 2:** Univariate and multivariate Cox regression analysis for overall survival

	univariate				multivariate	
			**initial model**		**final model**	

	**HR**	***p***	**HR**	***p***	**HR**	***p***

age>65 years	2.99	<0.0001	3.53	<0.0001	3.43	<0.0001

male sex	1.86	0.0018	1.84	0.0030	1.80	0.0038

ALC>14 × 10^9^/L	1.67	0.0062	1.34	0.17	-	

β_2_M>1 × ULN	1.43	<0.0001	1.51	0.06	1.59	0.029

LNR ≥3	3.06	<0.0001	1.20	0.59	-	

Binet stage		<0.0001				

A	ref		ref		ref	

B	3.20		2.19	0.0352	2.77	<0.0001

C	4.95		2.86	0.0028	3.68	<0.0001

Rai stage		<0.0001				

0	ref		ND			

I-II	1.66		ND			

III-IV	5.08		ND			

CD38>30%	1.73	0.0038	1.03	0.88	-	

ZAP-70>20%	1.53	0.0204	1.04	0.83	-	

Unmutated IGHV	2.46	<0.0001	2.04	0.0008	2.04	0.0003

del17p	3.44	<0.0001	2.14	0.0015	2.06	0.0022

del11q	1.39	0.248	ND			

Year of diagnosis		0.0130	(in model as strata)*		(in model as strata)*	

<2001	ref					

2001-2005	1.46					

>2005	2.34					

Therapy		0.106	ND			

Chemotherapy	ref					

Immunochemotherapy	0.6					

Final model obtained by stepwise backward elimination

HR: hazard ratio; ND: not done

**Table 3:** Prognostic score for overall survival with clinical and biological risk factors and bootstrap validation

	final model^1^			variable selection^2^	
	**β**	**HR**	***p***	**%**	**final score**

Age>65 years	1.23	3.43	<0.0001	100	2

Binet B	1.02	2.77	<0.0001	92	2

Binet C	1.30	3.68	<0.0001	92	3

Gender (male)	0.59	1.80	0.0038	94	1

β2M >1 × ULN	0.46	1.59	0.0294	66	1

Del17p	0.72	2.06	0.0022	93	1

Unmutated IGHV	0.71	2.04	0.0003	98	1

calendar year	(in strata)	-	-	87	-

HR: hazard ratio; β: Cox regression coefficient; ULN, upper limit of normal

^1 ^Final model adjusted for year of diagnosis

^2 ^Percentage of selection in 620 leave-one-out bootstrap samples. Percentages for del11, ZAP-70, CD38, LFN, ALC were respectively 28%, 21%, 15%, 24%, 46%

**Table 4:** Leave-one-out cross validation of the final model

Step	covariate removed	no. of variables	log-l	AIC	cvlog-l
0	all (null model)	0	-675.7	-675.7	-846.8

1	none (final model)	6	-502.1	-509.1	-743.1

2	β_2_M	5	-504.6	-510.6	-738.8

3	β_2_M and sex	4	-618.9	-623.9	-738.8

The final model included the following 6 variables: Binet stage, age > 65 years, unmutated IgVH, presence of del17p, male sex, β_2 _M > ULN

Log-l: log-likelihood, AIC: Akaike's information criterion (AIC); cvlog-l: cross-validated log likelihood Removal of the least important variables (beta2M and sex) in step 2 and 3 resulted in a reduction of the fit by at least 2 criteria, demonstrating the validity of the model with 6 variables (according to reference #26)

**Table 5:** Univariate and multivariate analysis of TTT in Binet A patients below 70 year of age (n = 291, no missing cases)

	univariate			multivariate			
				**initial model**		**final model**	

	**%**	**HR**	***p***	**HR**	***p***	**HR**	***p***

age>65 years	22	0.96	0.85	-			

male sex	56	1.01	0.97	-			

ALC>14 × 10^9^/L	42	1.35	0.0974	-			

β_2_M>1 × ULN	38	2.04	<0.0001	1.53	0.0242	1.64	0.0069

CD38>30%	21	2.46	<0.0001	1.42	0.1042	-	

ZAP-70>20%	36	2.72	<0.0001	1.39	0.1374	-	

Unmutated IGHV	32	3.60	<0.0001	2.16	0.0012	3.09	<0.0001

del17p	7	2.59	0.0015	1.37	0.31	-	

del11q	5	3.09	0.0005	2.21	0.02	1.97	0.0383

Year of diagnosis			0.85	-			

<2001	33	ref					

2001-2005	48	1.07					

>2005	19	0.89					

Final model obtained by stepwise backward elimination

HR: hazard ratio

### Clinical-biological prognostic index

**Figure 2:**
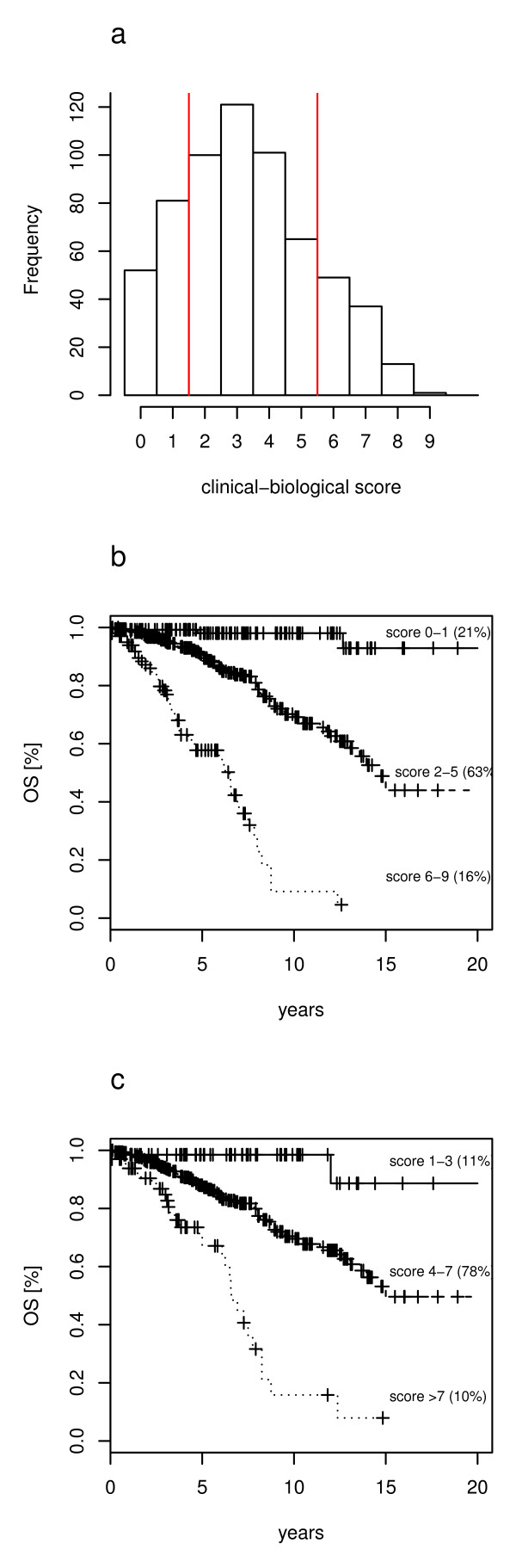
**Clinical-biological index**. **a**) histogram of score points according to clinical-biological prognostic model. Vertical red lines show the positions of cut points splitting sample in 3 risk groups. **b**) Kaplan-Meyer plot showing prognostic stratification in 3 risk groups according to clinical-biological score. **c**) prognostic stratification in 3 risk groups according to Wierda et al prognostic score^6^.

**Figure 3:**
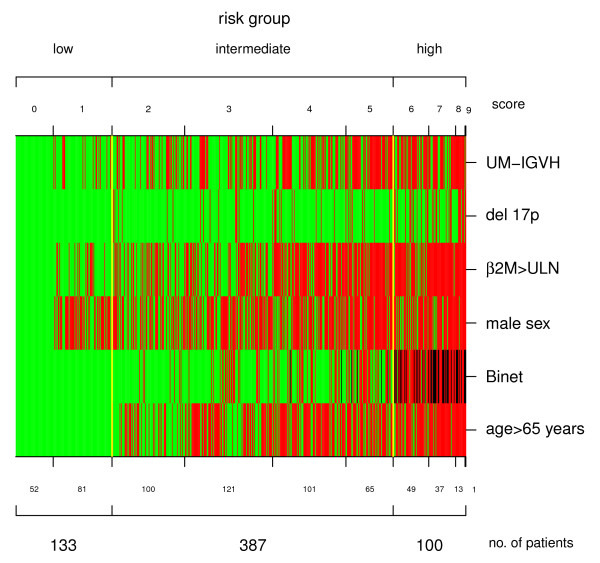
**Heatmap of individual patient clinical-biological scores**. Columns refer to individual patient; rows refer to predictors. In heatmap, each dicotomic predictor is indicated in green or red if present in its favourable or unfavourable configuration, respectively. Binet stages A, B, C are indicated in green, red and black, respectively. Yellow bars show the splits between low, intermediate and high-risk groups. Number of patients in each score class are reported at the bottom of columns.

**Figure 4:**
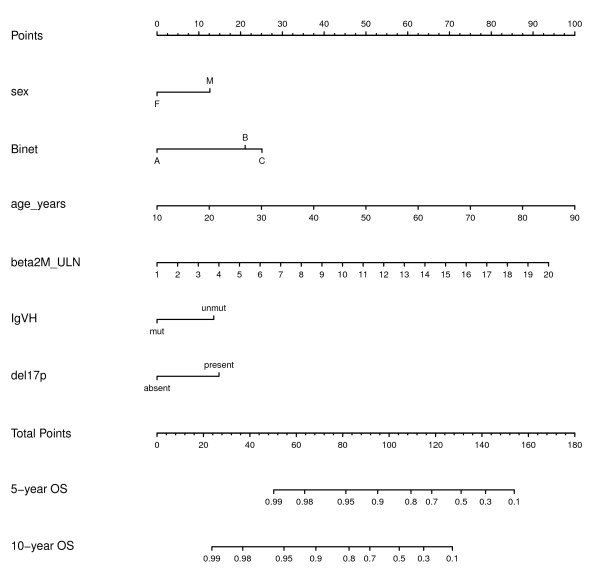
**Nomogram for predicting overall survival according to the clinical-biological prognostic index**. To read the nomogram, draw a vertical line from each tick marker indicating the status of a predictor to the top axis labeled Points. Sum the points and find the corresponding number on the axis labeled Total Points. Draw a vertical line down to the axes showing 5- and 10-year overall survival rates and median survival. Beta2M, ß2 microglobulin; ULN, upper limit of normal; OS, overall survival.

The 4-variable clinical model previously proposed by us [8] was refitted in the present CLL cohort (see Additional file 2: Table S1). The 4-variable clinical model had a greater discriminatory power (c-index 0.72) than the 6-variable clinical model by Wierda et al. (c-index 0.62; *p *= 1.3 × 10^-7^). According to the β regression coefficients, a novel clinical-biological prognostic score was developed by assigning 3 points for Binet C stage, 2 points/each for Binet B stage and age > 65 years, 1 point/each for male gender, high β_2 _M levels, presence of an unmutated IGHV gene status or 17p deletion (Table 3). The score point distribution is reported in Figure 2a. To this distribution we applied a recursive partitioning method [23], which yielded three prognostic groups, with score 0-1, 2-5 and 6-9. The Kaplan-Meier plots of the three risk group partitioning of the prognostic score is shown in Figure 2b, for comparison also the risk group partition by Wierda PI [3] is shown in Figure 2c. In particular, 21% of patients (score 0-1) were at low-risk, 63% (score 2-5) were at intermediate risk, and 16% of patients (score 6-9) were at high risk. Projected survival in respectively low, intermediate and high-risk groups was 98%, 90%, 58%, and 98%, 69% 9% at 5-year and10-year, respectively. Predictive accuracies were significantly greater in the clinical-biological model, compared to the 6-variable clinical model by Wierda et al. (c-index 0.73 vs 0.62, *p *< 0.0001) [3], or to the 4-variable clinical model (c-index 0.73 vs 0.72, *p *< 0.0001) [8], or to Binet (c-index 0.73 vs 0.65, *p *< 0.0001), or Rai (0.73 vs 0.62, *p *< 0.0001) staging systems. To show the combination of predictive variables in each patients and in each group we used a heat-map plot (Figure 3). In the low risk group, comprising 133 cases, 52 patients had no adverse predictors (score 0), 50 patients were male, 16 patients had a β2 M > 1 and 15 patients had unmutated IGHV gene mutational status. Of note, low risk patients were never aged >65, nor had a Binet staging B or C, or were affected by a CLL bearing del17p (Figure 4). Conversely, in the high-risk group, only 3 or 4 patients, respectively, had <65 years or a Binet stage A disease; these patients, however, had all the other prognosticators in their bad configuration. Moreover, the 51 patients in Binet stage B of the high-risk group, had mostly (37/51) an unmutated IGHV gene status or high β2 M (42/51) levels. Finally, the 29 patients classified in Binet stage C and belonging to the high-risk group, mostly had (26/29) high β2 M levels (Figure 4). Kaplan-Meyer plots of the individual variables are reported in Figure 4.

### Nomogram for estimating prognosis in individual patients

**Figure 5:**
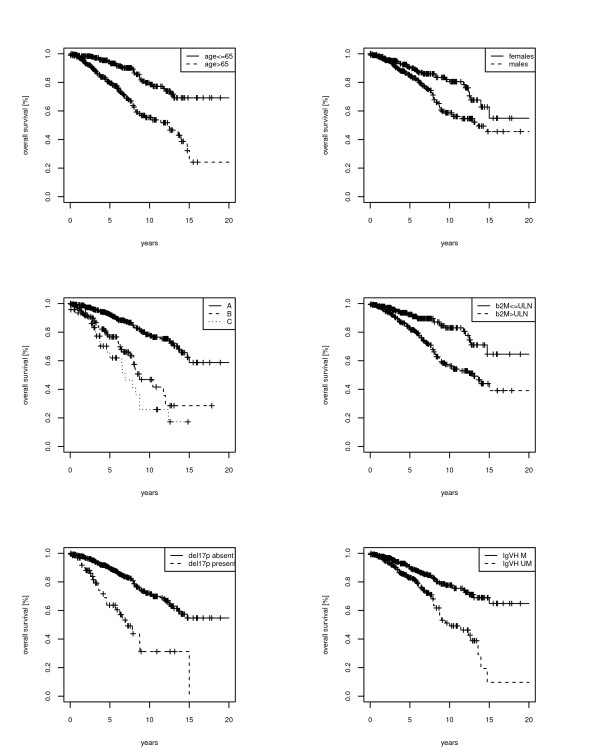
**Kaplan-Meyer plots of overall survival (OS) for the 6 variable of the clinical-prognostic index**.

Even if individual estimates of survival, as those obtained from nomograms, are more likely affected by inaccuracy than group estimates [28], to allow individual patients survival estimation a nomogram was developed as described previously [8], based on the final model with clinical and biological prognostic factors shown in Table 3, modified using age and β2 M as continuous variables (Figure 5). The clinical-biological nomogram showed a better predictive accuracy than the clinical nomogram proposed by Wierda et al. [3] (c-index respectively 0.79 and 0.76, *p *= 0.046).

## Discussion

Survival time at CLL diagnosis may be simply estimated by means of six variables, four of them clinical-demographic (stage, LNR, sex, age) plus two quantitative assays (ALC, β2 M) [3,4,8]. Two independent studies [4,8] failed to confirm the predictive power of ALC. A simplification of the PI from six to four variables was previously proposed by us as capable to stratify patients with equal or better performance [8]. In the present study, the aim was to improve these clinical prognostic models by adding information on biological variables, in particular those identified by the updated NCI-WG guidelines [1] as mandatory at least in the context of clinical trials. We demonstrated that PI for OS prediction based on clinical variables could be improved only by IGHV gene mutational status and del17p, but not CD38, ZAP-70 and del11q. The lack of prognostic power of CD38 and ZAP-70 is not totally unexpected. Similar findings have been found either analyzing OS [13,14,29] or TTT [30,31], although none of these reports included both biological and clinical prognosticators in a comprehensive clinical-biological PI, as proposed here. It has been often emphasized that assays evaluating ZAP-70 and, at least in part, CD38 expression suffer from inherent weakness and lack of proper standardization [13,32-34]. As a consequence, considerable analytic variability still exists on measurement of these parameters [35]. In this regard, such a variability could be more relevant in multi-center series like that investigated in this study. Indeed, at variance with our results, ZAP-70 or CD38 turned out to be among the strongest prognosticators in mono-center studies [36,37], with time-to-first-treatment or time-to-progression as end-points. Lack of reproducibility and standardization of biological markers can affect the results of prognostic tools applied at different institutions. Our model might be less subjected to this bias, since it includes IGHV and del17p, but not the less standardized measurements of CD38 and ZAP-70. Krober et al. [13,14] have previously showed the importance of molecular risk factors in CLL by stratifying patients by IGHV gene mutational status and presence of high-risk genomic aberrations (del17p or del11q), although authors failed to test if their model was independent of clinical and demographic risk factors. Here we had the chance to integrate the data by Krober et al. [13,14] by showing the independent prognostic relevance of UM IGHV gene status and del17p in a model that also included clinical and demographic risk factors. Of note, the effect of these molecular prognosticators was found to be additive and of equal importance. The unexpected limited relevance of molecular risk factors in our model and the lacking predictive power of CD38 and ZAP-70, may be in part justified by a relative small number or deaths and a median follow up of only 5 years, despite the large number of patients collected. Future analyses with longer follow-up data and more events might regain significance to some biological variables showing the need to update the score. Compared to our previous clinical model [8], we confirmed the value of β2 M, although with the smallest coefficient and the weakest level of significance. We had no data to adjust for renal function impairment, particularly in aged patients, or for other comorbidities. However, β2 M was shown to be important in other retrospective and prospective studies [38-40]. The value of prognostic factors in aged CLL patients has been recently criticized by showing that FISH aberrations (del11q or del 17q) and IGVH lost their predictive power for OS in patients aged above 75 years [41]. In our CLL series, we specifically addressed this issue by testing age dependent variations of the predictive power of all the variables included in the final model. No significant interaction effect was found for age. We found a greater proportion of death events in the oldest age group. The risk of death in this group may be influenced by other factors, not related to disease. However, epidemiological data from cancer registry show the frequent occurrence of late deaths attributable to CLL also in aged patients group [42,43]. The effect of chemoimmunotherapy with anti-CD20 was small, with a non significant trend for a longer survival (*p *= 0.10). Results of the present study differed in part from those of a randomized prospective trial [40], where Binet stage and gender, in addition to del11q, CD38 and ZAP-70, all failed to be independent prognostic markers in a multivariate model for OS which also included IGHV gene mutational status, usage of IGHV3-21 gene, del17p, age and β2 M. Notably, this study, which investigated a population of selected patients in need of treatment (i.e. with active or progressive disease), selected del17p as the strongest risk factor [40]. Conversely, in our retrospective study dealing with untreated patients at diagnosis, the relative weight of del17p appeared equal or lower than that of other variables. Therefore, while the model described in [40] seems to better predict outcome of CLL patients with progressive or active disease, our model appears to be more suited for estimating survival in untreated patients at diagnosis or before clinical progression. The analysis of TTT in Binet A patients below 70 years of age showed that demographic factors (age, gender), important for OS estimation, lost their prognostic power for TTT. Conversely, It might be expected that biological prognosticator, particularly those with limited or even absent significance in the OS analyses, would have gained more importance in the TTT analyses in this subset of patients. However only IGHV and β2 M confirmed their role, with IGHV the most important predictor of TTT. CD38 and ZAP-70 were again not significant, in spite of a good representation of positive cases (respectively 21% and 36%); del17p lost its power whereas del11q gained significance. In this case the low percentage of positive cases may, at least in part, justify the fluctuating results.

## Conclusions

In the present study we showed that the survival of untreated CLL patients may be estimated by a limited set of clinical and biological variables, integrated in a prognostic index and in a nomogram, allowing group and individual estimation, respectively. CD38, ZAP-70 and del11q gave redundant prognostic information. Both the proposed PI and the nomogram were only internally validated. Even in internally validated models, the performance of prognostic tools may be influenced or biased by the composition of the population in which they are developed and lack of standardization of biological variables. Therefore the prognostic tools proposed should be used with caution until externally validated on independent, prospective patient series.

## Abbreviations

ALC: absolute lymphocyte count; β2M: beta2-microglobulin; FISH: fluorescence in-situ hybridization; LNR: number of involved lymph node regions; HR: hazard ratio; IGHV: immunoglobulin heavy chain variable gene segments; OS: overall survival; PI: prognostic index; TTT: time to treatment; ULN: upper limit of normal.

## Competing interests

The authors declare that they have no competing interests.

## Authors' contributions

PB drafted the manuscript and performed statistical analysis, DR acquired data and helped to perform statistical analysis and to draft the manuscript, FF, GDP, FB, EZ, MM, GP, GDA, DE, RM and FL acquired and interpreted data and participated in the design of the study, GG, VG and LL participated in the design of the study and helped to draft the manuscript.

## Supplementary Material

Additional file 1 **Supplementary statistical methods**. Details of validation procedures for the whole prognostic model. Click here for file

Additional file 2 **Table S1**. Previously proposed prognostic score for overall survival with clinical risk factors. Click here for file
