# Supplementary material for: IGHV gene mutational status and 17p deletion are independent molecular predictors in a comprehensive clinical-biological prognostic model for overall survival prediction in chronic lymphocytic leukemia
Source: J Transl Med. 2012 Jan 30;10:18. doi: 10.1186/1479-5876-10-18 (PMC3297493; doi:10.1186/1479-5876-10-18)
Supplement: Additional file 1 — Supplementary statistical methods. Details of validation procedures for the whole prognostic model. [file 1479-5876-10-18-S1.PDF]

Supplementary statistical methods: details of the validation procedures.

1) Predictors selection.

Initial selection was performed by step-down deletion from the full Cox model using the Akaike criterion (AIC) to discard variables. Bootstrap was used subsequently as a validation tool. We used the R function "validate" from package Design. This function allows to replicate the predictor's selection process and to estimate optimism using a .632+ method. In particular: a) we used the .632+ bootstrap method, with B=620; b) to avoid any preselection bias, we included all candidate variables, even if not significant in univariate analysis c) a step-down selection (by AIC) was performed in each bootstrap sample, to replicate exactly the building model procedure; 7 variables, selected in more than 50% of cases, were included in the final model. Prediction error corrected for optimism (estimated by .632 bootstrap method) was 0.244.

2) Model refitting on the selected predictors

This step was validated following a cross validation approach (ref #26). An additional validation procedure to estimate the overfitting of the final model was done again using the R function "validate" from package Design, with the bootstrap .632+ method. The prediction error, corrected for optimism was 0.247.

3) Selected predictors score assignments

To reduce at a minimum arbitrary rules in score assignments, we made use only of rounding (performed by a software function) and division. We followed in this a standard procedure (M Parmar, D Machin: Survival Analysis. A practical approach. Wiley 1995. Chapter 9: Prognostic Indices). First we rounded the decimal part of β regression coefficients at the second digits. Then we divided all β coefficients by 0.50. This value was (arbitrary) chosen by rounding the β2m β coefficient (original value: 0.46, the smallest in the model). Finally we rounded to the nearest integer. Individual patient score were obtained by summing these integers, conditional on the presence of the corresponding unfavorable predictor variable. The variability introduced by score assignment turned out negligible (see below).

4) risk groups assignment with prognostic score cut-points found by recursive partitioning

Recursive partitioning was used to split sample in risk groups based on score. Prediction error for this step was estimated in two ways. First, to check misclassification errors introduced by the score assignment process, we applied recursive partitioning directly to linear predictors from final model. The classification was in agreement in 98% of cases, showing that very little error was introduced. Second, prediction errors introduced by random partitioning was analyzed by a "random forest" procedure (ref#27). We used the R package randomSurvivalForest, drawing n=1000 bootstrap samples, growing n=1000 trees, predicting survival in each individual by a leave-one-out procedure, and then estimating prediction error by 1-C, where C is Harrel's concordance index. Estimated prediction error was 0.29.

5) Whole procedure cross validation

Prediction error in the final categorical risk model was estimated by a comprehensive leave-one-out cross validation. In each iteration one sample was omitted, and the whole process of model building performed in the (n-1) sample. In particular, in the (n-1) sample we performed variable model selection (step 1), refitting of selected variables (step 2), score assignment by rounding and division (step 3). We then predicted the score of the omitted sample. This was repeated for all samples, resulting in a cross-validated score. Finally we split sample in risk groups according to recursive partitioning applied to the cross-validated score (step 4). Therefore, the risk group was assigned to each case using a model that did not use that case in model building. Prediction error was estimated 1) as misclassification error, with a confusion matrix between cross validated risk score and original risk score. 2) by estimating prediction error as 1-C, where C is is Harrel's concordance index. Estimated prediction error was 0.29. Misclassification error was 9%.
