# Supplementary material for: IGHV gene mutational status and 17p deletion are independent molecular predictors in a comprehensive clinical-biological prognostic model for overall survival prediction in chronic lymphocytic leukemia
Source: J Transl Med. 2012 Jan 30;10:18. doi: 10.1186/1479-5876-10-18 (PMC3297493; doi:10.1186/1479-5876-10-18)
Supplement: Additional file 2 — Table S1. Previously proposed prognostic score for overall survival with clinical risk factors. [file 1479-5876-10-18-S2.PDF]

Table S1. Prognostic score for overall survival with clinical risk factors

	Clinical model			
	β	HR	*p*	score
Age>65 years	1.16	3.21	<0.0001	2
Binet B	1.23	3.43	<0.0001	2
Binet C	1.53	4.62	<0.0001	3
Gender (male)	0.65	1.92	0.0012	1
β2M >1xULN	0.52	1.68	0.0127	1

HR: hazard ratio; β: Cox regression coefficient; ULN, upper limit of normal
